# A New Measurement Method for High Voltages Applied to an Ion Trap Generated by an RF Resonator

**DOI:** 10.3390/s21041143

**Published:** 2021-02-06

**Authors:** Yunjae Park, Changhyun Jung, Myeongseok Seong, Minjae Lee, Dongil Dan Cho, Taehyun Kim

**Affiliations:** 1 Department of Electrical and Computer Engineering, Seoul National University, Seoul 08826, Korea; yjpark0128@snu.ac.kr (Y.P.); chjung91@snu.ac.kr (C.J.); mjlee88@snu.ac.kr (M.L.); dicho@snu.ac.kr (D.D.C.); 2 Automation and Systems Research Institute, Seoul National University, Seoul 08826, Korea; 3 Inter-University Semiconductor Research Center, Seoul National University, Seoul 08826, Korea; 4 Department of Mechanical Engineering, Seoul National University, Seoul 08826, Korea; mss8087@snu.ac.kr; 5 Department of Computer Science and Engineering, Seoul National University, Seoul 08826, Korea; 6 Institute of Computer Technology, Seoul National University, Seoul 08826, Korea

**Keywords:** ion trap, voltage divider, RF voltage, helical resonator

## Abstract

A new method is proposed to measure unknown amplitudes of radio frequency (RF) voltages applied to ion traps, using a pre-calibrated voltage divider with RF shielding. In contrast to previous approaches that estimate the applied voltage by comparing the measured secular frequencies with a numerical simulation, we propose using a pre-calibrated voltage divider to determine the absolute amplitude of large RF voltages amplified by a helical resonator. The proposed method does not require measurement of secular frequencies and completely removes uncertainty caused by limitations of numerical simulations. To experimentally demonstrate our method, we first obtained a functional relation between measured secular frequencies and large amplitudes of RF voltages using the calibrated voltage divider. A comparison of measured relations and simulation results without any fitting parameters confirmed the validity of the proposed method. Our method can be applied to most ion trap experiments. In particular, it will be an essential tool for surface ion traps which are extremely vulnerable to unknown large RF voltages and for improving the accuracy of numerical simulations for ion trap experiments.

## 1. Introduction

An ion trap is an essential physical platform in quantum information processing including quantum computing [1,2,3,4,5] and quantum networks [6], attributed to its high-fidelity quantum gates [7,8] and long coherence time [9]. Ions are confined by potentials created using electric fields, which allow fine control of various parameters of the potential [10,11,12,13,14]. To create sufficient potential to trap ions, a Paul trap requires strong oscillating electric fields that are generated by high voltages applied to the radio frequency (RF) electrode of the trap [15]. Generally, resonators such as RLC resonant circuits [16] or helical resonators [17] are used as RF voltage amplifiers to generate the high voltages necessary for the ion trap. However, the voltage gain of the RF resonator is extremely sensitive to resistance changes and load or resonator capacitance. Therefore, only the order of magnitude of voltage gain can be inferred to be proportional to the square root of the quality factor (Q) based on the law of conservation of energy [18,19]:

(1) $$V_{\mathrm{OUT}}=\eta \sqrt{Q}V_{\mathrm{IN}},$$

where $V_{\mathrm{IN}}$ and $V_{\mathrm{OUT}}$ are RF amplitudes of input and output voltages, respectively, and η is a geometrical factor of the helical resonator.

As a result of the uncertainty in the voltage gain estimation, the absolute RF voltage applied to the ion trap can only be determined by comparing the measured secular frequencies with secular frequencies estimated by a numerical simulation. This approach has a serious flaw, in that the absolute RF voltage cannot be determined until the first ion is trapped and the secular frequencies of the trapped ion are measured, although this voltage is one of the most important factors for successful ion trapping. However, this method is used by most ion trap experiments as there is no alternative, and generally the proper voltage for trapping condition is searched for until the first ion is trapped. During the search, occasionally, an RF voltage higher than the breakdown voltage is applied to the RF electrodes and breakdown occurs, but this was not considered a serious problem for a macroscopic trap because there was little damage to its structure.

**Figure 1:**
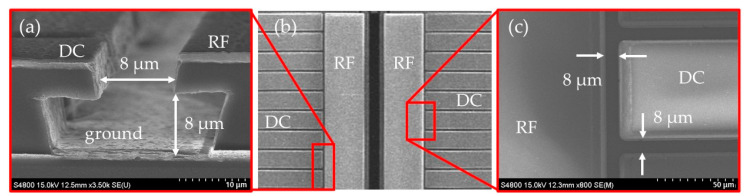
Examples of small gaps between the radio frequency (RF) electrode and adjacent electrodes. (**a**) Cross-section of a surface trap, (**b**) typical layout of electrodes in a surface ion trap, and (**c**) typical gaps between electrodes. All scanning electron microscope (SEM) pictures were taken of the surface ion trap introduced in reference [27].

Conversely, as ion traps attract more attention for their potential in a scalable quantum computers, more research has been focused on surface ion traps [2,8,20,21,22,23,24]. However, the distance between the RF electrode and an adjacent electrode in a typical surface trap is less than 10 µm [25,26,27], as shown in Figure 1, and an unknown high voltage applied to a surface ion trap can cause permanent damage to a chip if it exceeds a certain level [28,29].

Therefore, as surface ion traps become more popular, it is critical to measure the absolute amplitude of RF voltages amplified by helical resonators. Unfortunately, the extreme sensitivity to the load impedance of the voltage gain of the helical resonator prevents the use of typical RF voltage measurement equipment such as oscilloscopes, power meters, or spectrum analyzers. Gandolfi et al. [16] proposed a method to infer the amplitude of RF voltages amplified by an RF resonator using a capacitive voltage divider. Johnson et al. [10] proposed a method to stabilize RF voltages applied to the trap by measuring the voltage with a capacitive voltage divider installed inside a helical resonator, which has been used in many other experiments [11,12,13]. However, without proper shielding, the dividing ratio of practical capacitive voltage dividers is easily affected by environmental coupling. For example, Detti et al. [30] showed linearity in measured values by using multiple capacitive voltage dividers, but the absolute values of the RF voltages could not be measured. These couplings add an uncontrolled amount of capacitance to the circuit, and the dividing ratio of the voltage divider at resonant frequencies becomes unpredictable, as discussed in Section 3.1. Using the existing methods [10,16], the absolute value of RF amplitude cannot be measured, and only relative changes with respect to some unknown constant voltage can be measured. Of course, the measurement of the relative changes is sufficient to keep the secular frequency constant, as shown in reference [10]. However, because the actual amplitude of the RF voltage is still unknown, the voltage gain of the helical resonator must be inferred by comparing the measured secular frequency and numerical simulation results. Therefore, even with these approaches using capacitive voltage dividers, the absolute voltage of the RF amplitude remains unknown until the secular frequency can be measured with trapped ions, and there is a potential threat to cause damage to a surface trap. For metrology purposes, it is also very important to model the electrical properties of the combined helical resonator and trap [31]. Apriyana et al. [32] designed an RF circuit to apply correct voltages to ion traps. However, modeling equivalent RF circuits including ion traps is difficult and the applied voltages do not match those designed by the model.

To overcome these limitations, we propose to use a voltage divider module with proper RF shielding and SubMiniature version A (SMA)-type input and output ports. The frequency response of the shielded divider module is first characterized without a helical resonator and then connected to the monitoring port of the helical resonator to measure the output voltage of the divider. Finally, based on the pre-calibrated dividing ratio at a specific frequency, the measured output voltage can be converted to the output voltage of the helical resonator. To verify that our method can provide correct values for the absolute amplitude, we first obtained the functional relation between the measured secular frequency ($\omega_{i}$) and the measured amplitude ($V_{T}$) of the RF voltage applied to the trap using the voltage divider. Then, the same relation between $\omega_{i}$ and $V_{T}$ was found using the numerical simulation. Finally, we confirmed that the measured functional relation and the prediction from the numerical simulation are within 13% of each other in the worst case.

Section 2 provides a vacuum chamber system with an ion trap, a summary of the numerical simulation method, and the design of a voltage divider. In Section 3, the characterization result of the voltage divider and modification of the helical resonator are provided, and the measurement result and the simulation results are compared.

## 2. Experimental Background

### 2.1. Surface Ion Trap and Vacuum Chamber

**Figure 2:**
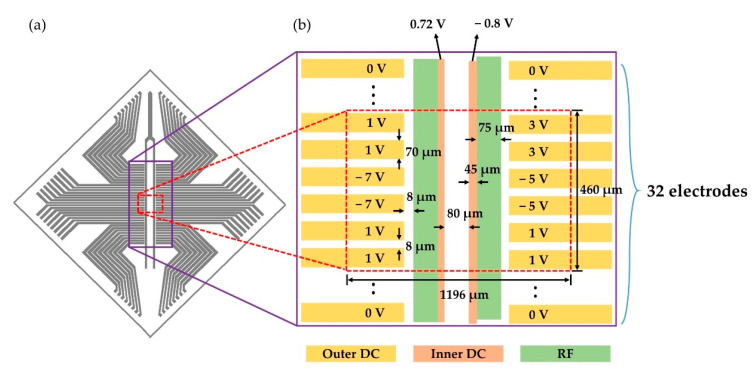
Comparison of the electrode layout of the entire surface ion trap chip and the numerical simulation. (**a**) Layout of the top layer electrodes in the surface trap used for the measurement. (**b**) Dimension of the electrodes near the trapping region (not drawn to scale) and the DC voltages used in our simulation. Inner DC electrodes are 14 μm below the RF and outer DC electrodes. The dashed red box indicates the electrode geometry used in the numerical simulation. Note that the red box shows only a small part of the entire chip.

A typical chamber for an ion trap experiment is composed of pumps for ultra-high vacuum, viewports, feedthroughs for DC and RF voltages, and an ion trap structure [33,34]. We use a Paul trap in the shape of a surface ion trap [35,36] and mount it in a vacuum chamber with a pressure of approximately 2 × 10^−11^ Torr at room temperature. A high RF voltage amplified by a helical resonator is applied to the ion trap through the RF feedthrough. A Paul trap is composed of RF and DC electrodes, which can be arranged in various ways [15,29,37,38]. Figure 2a shows the layout of the RF and outer DC electrodes in the top layer of the chip used in our experiment. Inner DC electrodes are 14 µm below the top layer. Figure 2b shows the dimensions of the electrodes around the trapping region, with DC voltages used in the experiment and the numerical simulation. The dashed red box shows the electrode geometry used in the simulation; further details are discussed in Section 2.2.

**Figure 3:**
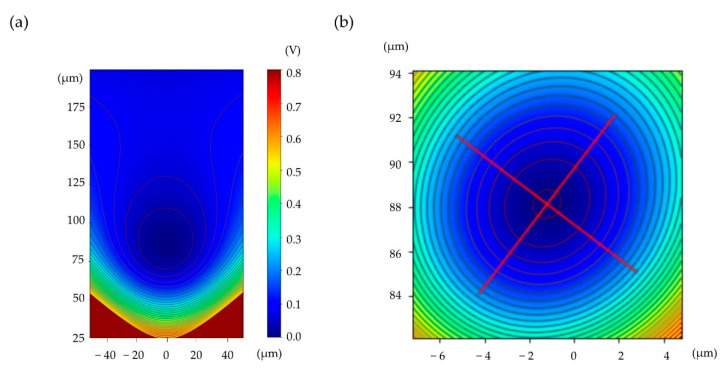
Example contour plot of total potential obtained by numerical simulation of the electrode geometry shown in the dashed red box of Figure 2b. (**a**) Example of total potential generated by voltages on DC and RF electrodes when the amplitude of the RF voltage ($V_{T}$) is 140 V and the frequency is 22.2 MHz which is the same as the experimental condition. DC voltages are shown in Figure 2. (**b**) Magnified view of (**a**) around the potential minimum. Two principal axes of the total potential are plotted at the null point. These two principal axes are intentionally tilted for efficient Doppler cooling. The third principal axis is orthogonal to these two axes and is not shown in this plot.

The minimum point of the pseudopotential [15] generated by the oscillating voltage on the RF electrodes appears around 90 μm above the surface of the chip, as shown in Figure 3. The total potential is determined by the sum of the pseudopotential and the static potential created by voltages applied to the multiple DC electrodes, as shown in Figure 2b. The equipotential surfaces near the trap position can be approximated as ellipsoids with three principal axes. RF voltage amplitude directly controls pseudopotential strength, and because secular frequencies of the trapped ion are determined by curvatures of the three-dimensional potential along these principal axes, they are functions of the RF voltage amplitude, the absolute value of which we want to measure in this work. The functional relation between secular frequencies and absolute voltages can be predicted by the numerical simulation, as discussed in Section 2.2. Conversely, the applied voltages and the corresponding secular frequencies can be measured experimentally, as discussed in Section 3.2 and Section 3.3, respectively. Therefore, the predicted and measured secular frequencies will be used to compare voltages used in the numerical simulation and absolute voltages measured by the method proposed in this work.

### 2.2. Numerical Simulation

We use a numerical simulation to analyze the relationship between voltages applied to the ion trap and secular frequencies of the trapped ion. To this end, we first calculate the electric fields formed around the ion trap when a voltage of 1 V is applied to only one of the electrodes, and the other electrodes are set to ground. Then we find $E_{RF}\left(r\right)$ by adding all the electric fields calculated for all the RF electrodes and scaling it by the RF voltage amplitude ($V_{T}$), where r represents the position vector. Finally, the pseudopotential is found by [35]

(2) $$\phi_{\mathrm{pp}}\left(r\right)=\frac{e^{2}{\left|E_{\mathrm{RF}}\left(r\right)\right|}^{2}}{4m\Omega^{2}},$$

where e is the charge of the ion, m is the mass of the ion, and Ω is the radio frequency of the voltage applied to the RF electrodes. The static potential can be calculated by linear combination of the other electric fields generated by all DC electrodes, and the total potential ϕ(r) can be obtained by adding the pseudopotential ($\phi_{\mathrm{pp}}\left(r\right)$) and the static potential. Figure 3a shows a contour plot of total potential generated by voltages applied to the electrodes of the surface ion trap, with layout and DC voltages, as shown in Figure 2b. For the RF voltage, $V_{T}=140\;V$ and Ω=2π⋅22.2 MHz are assumed. Figure 3b is a magnified view of the total potential around the minimum point with the two principal axes.

Based on the total potential ϕ(r) calculated by the numerical simulation, the secular frequencies ($\omega_{i}$) can be obtained by

(3) $$\omega_{i}=\frac{e}{m}\frac{d^{2}\phi \left(r\right)}{dr_{i}^{2}},\;\;i=1,2,3,$$

where $r_{i}$ are the new coordinates along the three principal axes.

**Figure 4:**
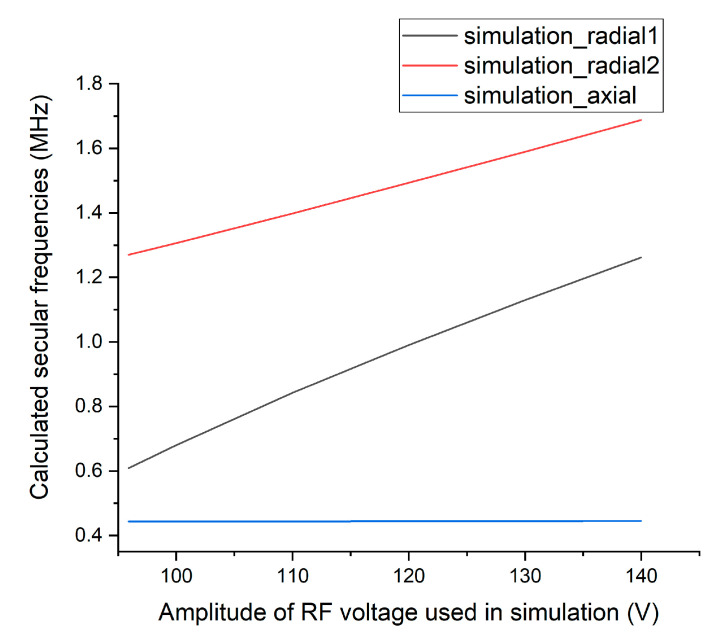
Expected secular frequencies of a ^174^Yb^+^ ion trapped by a total potential created by the RF voltage applied according to the numerical simulation. All the DC voltages remain constant as shown in Figure 2b.

Figure 4 shows the expected secular frequencies of a ^174^Yb^+^ ion trapped by a total potential created by the RF voltage applied to the trap, according to the numerical simulation. Our surface trap is a variant of a linear RF trap [15], where two secular frequencies along the principal radial axes in the transverse plane strongly depend on RF field strength. Conversely, the RF field should not affect secular frequency along the longitudinal axis in the ideal linear trap. Figure 4 shows that secular frequencies along the two radial directions change almost linearly with RF voltage amplitude, whereas the secular frequency along the axial direction barely changes.

For an accurate numerical simulation, the entire area of the ion trap shown in Figure 2a must be considered, but this is practically very difficult because of the limitations of the simulation tools. Therefore, we use only a part of the ion trap for the simulation. The dashed red box in Figure 2b shows the geometry of the electrodes included in our numerical simulation, which is a very small part of the entire chip. This limitation causes some uncertainty in numerical simulation results. Depending on electrode geometry and simulation tools, numerical simulation accuracy might be improved, but generally, surface traps have very thin electrodes, which require a careful arrangement for numerical simulations. Therefore, some discrepancy between the numerical simulation and experimental results is expected.

### 2.3. Voltage Divider Design

**Figure 5:**
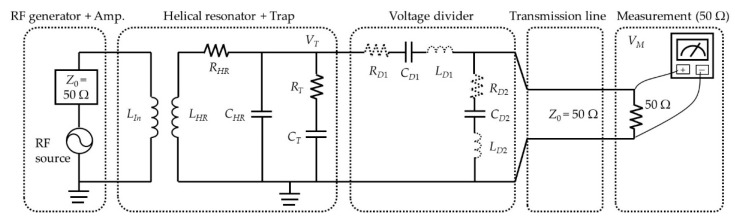
Equivalent RF circuit model of our ion trap setup with a helical resonator, a trap, and measurement setup for the RF voltage amplified by a helical resonator using a voltage divider.

The main role of the helical resonator in an ion trap setup is to amplify a small input RF voltage to the large amplitude required to trap an ion. Unfortunately, the voltage gain of the helical resonator decreases as the load capacitance connected to its output increases. The largest contribution to the load of the helical resonator generally comes from the ion trap by itself. Because the voltage divider will be connected to the helical resonator output in parallel with the ion trap, the contribution of the equivalent capacitance posed by the voltage divider to the total load capacitance should be very small compared with that of the trap, to avoid significant voltage gain reduction [16]. Figure 5 shows a simplified circuit model for our experimental setup with an RF voltage source including an RF amplifier, a helical resonator connected to a trap, a voltage divider, and a measuring instrument [39]. *L_In_* and *L_HR_* are the inductance of the input coil and the main coil of the helical resonator, respectively. *R_HR_* and *R_T_* are the parasitic resistance of the helical resonator and the ion trap, respectively. *C_HR_* and *C_T_* are the capacitance of the helical resonator and the ion trap, respectively. *R_D_*_1_ and *R_D_*_2_ are the sums of the contact resistance and the equivalent series resistance (ESR) of the capacitors in the voltage divider, and *L_D_*_1_ and *L_D_*_2_ are the equivalent series inductance (ESL) of the corresponding capacitors.

The impedance of the voltage divider (*Z_D_*) equipped with a measuring instrument with input impedance of $Z_{0}=50\;\Omega$ can be expressed as follows:

(4) $$Z_{D}=R_{D1}+\frac{1}{j\omega C_{D1}}+j\omega L_{D1}+\left(R_{D2}+\frac{1}{j\omega C_{D2}}+j\omega L_{D2}\right)\;||\;Z_{0}.$$

The voltage measured by the measuring instrument is

(5) $$\;V_{M}=\;\frac{\left(R_{D2}+\frac{1}{j\omega C_{D2}}+j\omega L_{D2}\right)\;||\;Z_{0}}{Z_{D}}V_{T},$$

where $V_{T}$ is approximately the same as the voltage applied to the trap because $R_{T}$ is negligible compared to the impedance of the $C_{T}$ at the applied RF frequency.

For the voltage divider, capacitors with very low ESR and ESL are chosen, and in the following discussion, we ignored ESR and ESL in our approximation because they are negligible compared to the impedance of the capacitance *C_D_*_1_ and *C_D_*_2_ within the frequency range used in a typical ion trap setup. For the selection of capacitance *C_D_*_1_ and *C_D_*_2_, there are two constraints we need to consider: First, the dividing ratio should not change much even when a measuring instrument with input impedance $Z_{0}$ is connected. The other constraint is that the equivalent capacitance of the voltage divider should be very small compared to the capacitance of the trap to avoid increasing the load of the helical resonator.

To satisfy the first constraint, the impedance of $C_{D2}$ should be negligible compared with the typical characteristic impedance of $Z_{0}$, requiring that $\left|1/\left(\omega C_{D2}\right)\right|\ll 50\;\Omega$ [16]. Therefore, when $C_{D2}$ is at least 1 nF around ω≈2π⋅20 MHz, *Z_D_* can be simplified as follows:

(6) $$Z_{D}\approx \frac{1}{j\omega C_{D1}}+\frac{1}{j\omega C_{D2}}.$$

The typical capacitance of our trap is $C_{T}\approx 10\;\mathrm{pF}$, and by choosing $C_{D1}\approx 1\;\mathrm{pF}$ (0603J2501P00BUT, Knowles) and $C_{D2}\gg C_{D1}$ in Figure 5 to satisfy the second constraint, the total capacitance of our voltage divider becomes less than 1 pF, causing little change in the load impedance. Therefore, any capacitance $C_{D2}$ larger than 1 nF can satisfy the two constraints. However, if we choose $C_{D2}\gg 1\;\mathrm{nF}$, the output of the divider becomes too small and the inaccuracy of the measurement increases. Hence, we used $C_{D2}\approx 1\;\mathrm{nF}$ (500S42E102KV4E, Johanson Technology) to satisfy both constraints around ω≈2π⋅20 MHz. 

**Figure 6:**
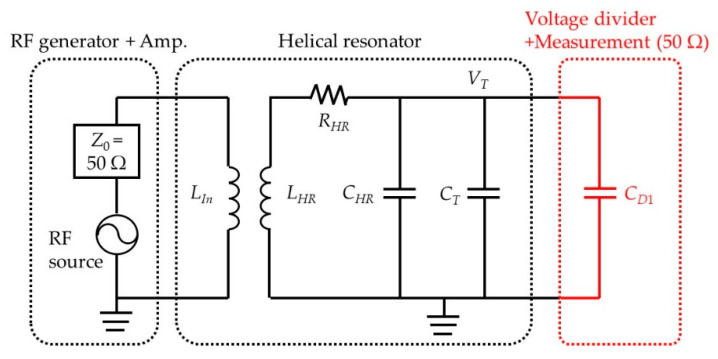
Simplified equivalent RF circuit model for our ion trap setup.

Figure 6 shows the simplified experimental RF circuit model, where *R_T_* is also omitted because of its small size.

The divided voltage (*V_M_*) measured by the measuring instrument is approximately as follows:

(7) $$V_{M}\approx \frac{1/\left(j\omega C_{D2}\right)\;\;}{Z_{D}}V_{T}\approx \frac{C_{D1}}{C_{D1}+C_{D2}}V_{T}\approx \frac{1}{1000}V_{T}.$$

Thus, the amplitude of the RF voltage measured by the voltage divider is approximately 0.001 times that applied to the trap.

## 3. Experimental Methods and Results

### 3.1. Voltage Divider Characterization

Based on Section 2.3, we can simply make a voltage divider that can allow us to measure large voltages applied to the RF electrodes with the dividing ratio of 1:1000. Unfortunately, a capacitor at radio frequency generally cannot be modeled as a simple capacitor because of ESR. Moreover, capacitors with very small capacitance are extremely sensitive, attributed to parasitic capacitance caused by environmental coupling. Therefore, developing a voltage divider with a specific dividing ratio is especially when the voltage divider includes a 1 pF capacitor. 

**Figure 7:**
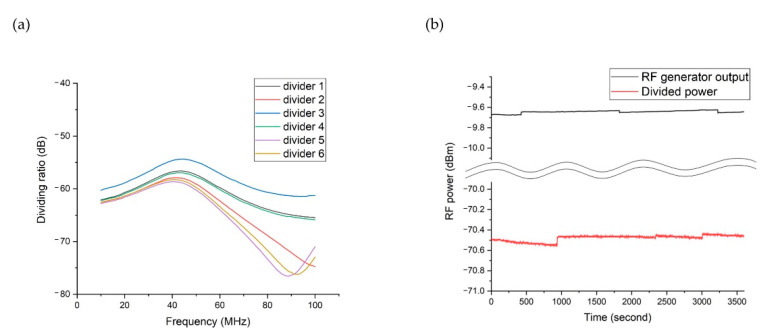
Characterization of voltage divider modules. (**a**) Measured dividing ratios of six voltage dividers as a function of input frequency (input power = −10 dBm). (**b**) Measured RF power of an RF generator output and output of voltage divider 1 over 1 h (input signal = 22.2 MHz, −10 dBm). RF generator output and divider power were measured at different times, but they were plotted in the same plot to show the time scale of RF power measurement drift. The standard deviation of the divided power is 0.028 dB.

To minimize unpredictable coupling to different environments, we developed modularized voltage dividers using a shielded enclosure (SMA-KIT-1.5MF, Crystek Corporation) with integrated SMA input and output shielding. To measure the voltage dividing ratio, we applied an RF voltage with a calibrated power source (SG384, Stanford Research Systems) to the input of the voltage divider, measured the output power with a spectrum analyzer (N9914A, Keysight), and plotted the power reduction in dB at each frequency. Figure 7a shows the frequency responses of the six different voltage dividers we built using the same components. As shown in Figure 7a, most of the voltage dividers had around −60 dB reduction s, corresponding to the expected 1:1000 voltage ratio at lower frequency range; however, as the frequency increased, the voltage ratio showed strong frequency dependence. Conversely, the measured voltage ratios of the unshielded voltage dividers exhibited strong frequency dependence from much lower frequency ranges. Even worse, the measurement result was not repeatable, and the unshielded voltage dividers showed different frequency responses even under the same measurement environment.

Figure 7a shows that these voltage dividers have resonant frequencies that are much lower than the self-resonant frequency of each capacitor, and explanation of these resonances requires full electromagnetic field analysis of the circuit. However, based on the large variations among the different voltage dividers as shown in Figure 7a, we can conclude that such kind of complicated analysis still cannot provide an accurate model. Therefore, instead of trying to model the complex behavior, we propose calibrating the dividing ratios for typical frequency range used by ion trap experiments with a stable RF source and spectrum analyzer first and then using the calibration data to measure the actual high RF voltage. We also evaluated whether the dividing ratio of the shielded voltage divider might drift over a long period of time. Figure 7b shows the measured long-term drift of RF generator output and also the measured long-term drift of voltage divider output when the same RF generator output is applied. These measurements were made at different times because the same spectrum analyzer needed to be used to make a fair comparison. From the RF generator output measurements, we can see that the major drift in the divider output measurements comes from either the RF generator or the spectrum analyzer, or both. A few discontinuities in the plot originate from the self-calibration feature of the spectrum analyzer. We also extended the evaluation period up to 4 days and confirmed that there is no further drift. Finally, we checked the impact of environmental change, and it is insensitive to relative humidity between 25% and 60% at the room temperature, but for the temperature, we observed +0.01 dB/°C between 25 °C and 55 °C.

### 3.2. Construction of the Divider and the Helical Resonator 

**Figure 8:**
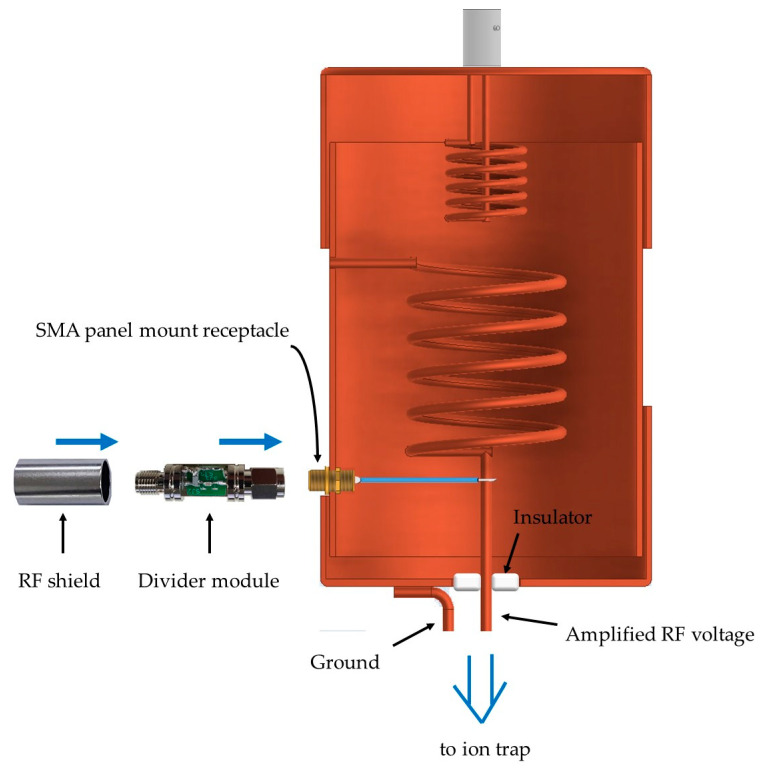
Construction of the shielded voltage divider and the helical resonator with a monitoring port. The voltage divider is connected to the helical resonator through a shielded SubMiniature version A (SMA) receptacle.

The helical resonator used in our test has a resonant frequency of 22.2 MHz when our trap is connected [17]. Although the voltage dividers developed in this work will have parasitic impedances not properly estimated in our simple model as shown in Figure 7a, the frequency response will not change as long as the requirements discussed in Section 2.3 are satisfied because the relation between the input and output of the modularized voltage divider is characterized after the divider is shielded. To probe the output of the helical resonator, we installed an SMA panel mount receptacle to it, as shown in Figure 8. Although Figure 8 shows the RF shield of the voltage divider module separately to visualize how the divider module is constructed, the RF shield of the module is fixed mechanically to the body of the module before all the characterization is performed.

### 3.3. Measurement of Secular Frequencies

**Figure 9:**
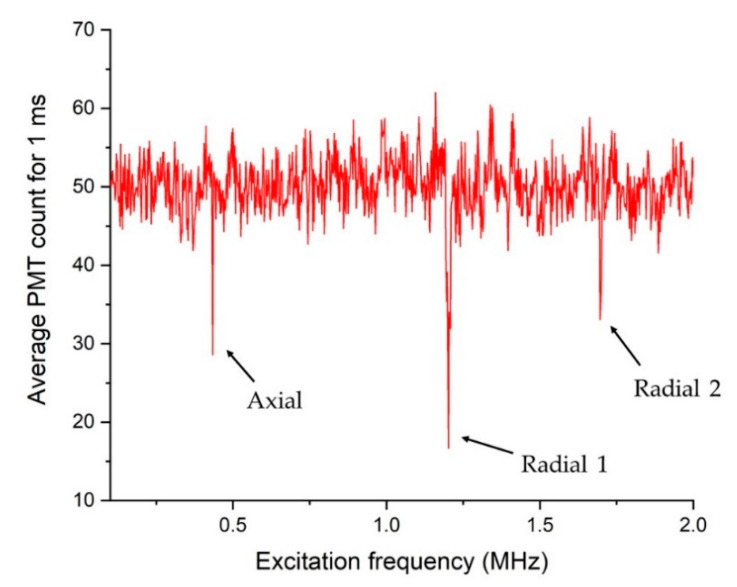
The number of photons emitted by trapped ions as a function of excitation frequency.

The secular frequency of a trapped ion can be measured by monitoring the amplitude change of the harmonic oscillation with excitation at the resonant frequency. Excitation with some specific frequency can be achieved either by amplitude modulation of the RF voltage applied to the ion trap [40] or by direct application of a small voltage oscillating at the secular frequency to one of the DC electrodes. In this work, we used both methods to cross-check our measurement results. As we scan excitation frequencies, the amplitude of secular motion starts to increase only when the excitation frequency coincides with the secular frequency, and a photomultiplier tube (PMT) can detect a change in the number of photons emitted from the ions [40]. Using this method, the secular frequencies of trapped ^174^Yb^+^ ions are measured. Figure 9 shows an example of the number of photons emitted from trapped ions as a function of the excitation frequency. We observed abrupt changes in the amount of photons at three frequencies, which correspond to the axial and radial frequencies of our trapping potential as was discussed in Section 2.2.

### 3.4. Relation between Measured Voltages and Measured Secular Frequencies

**Figure 10:**
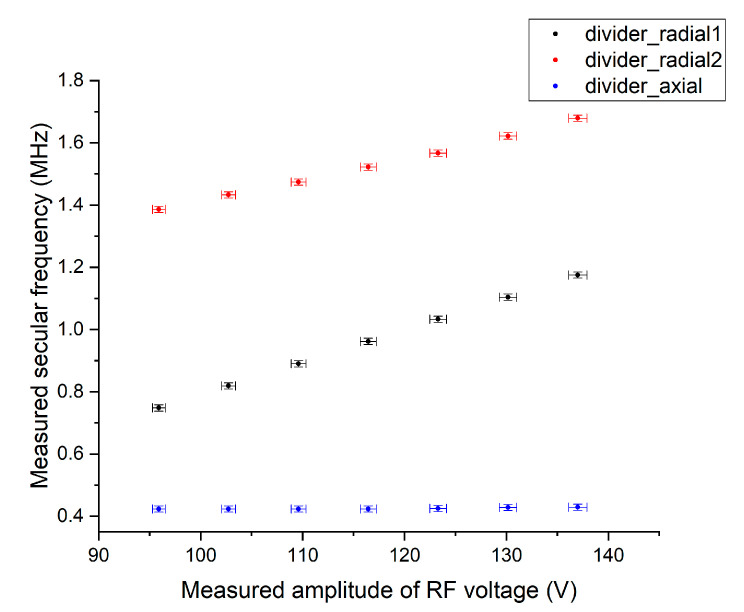
Relation between measured secular frequencies and RF voltage amplitude measured by the proposed voltage divider. The uncertainty of the measured RF voltage amplitude is 0.659%**.**

When an unknown constant RF voltage is applied to the ion trap through a helical resonator, secular frequencies can be measured using the method in Section 3.3, and the amplitude of the corresponding RF voltage can be measured using the voltage divider explained in Section 3.2. Figure 10 shows measured secular frequencies as a function of measured RF voltage.

**Figure 11:**
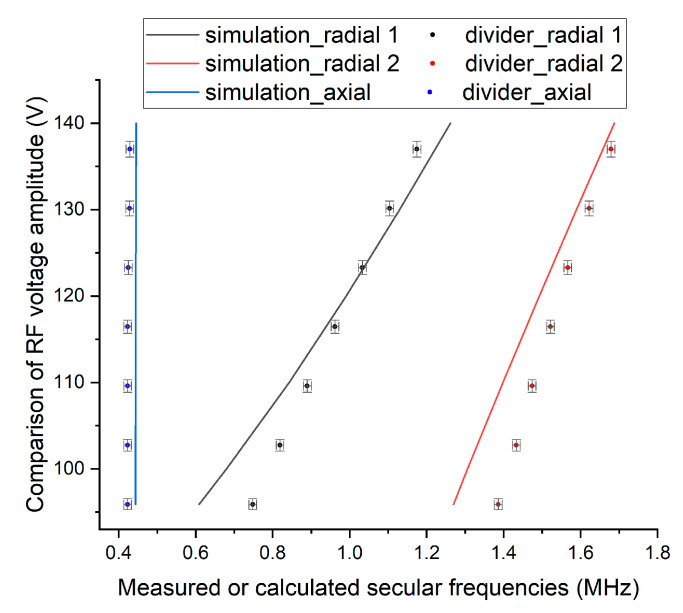
A comparison of RF voltage measured by the voltage divider with the estimated voltage necessary to obtain the same secular frequencies based on the numerical simulation. The relation between estimated voltage and expected secular frequencies comes from Figure 4.

In Figure 11, we compare the RF voltage measured by the voltage divider with the voltage necessary to obtain the same numerical secular frequencies as the measured secular frequencies that was estimated by the numerical simulation discussed in Section 2.2. Figure 11 shows that most of the expected voltages are very close to the absolute values of the measured voltage, which demonstrates the reliability of this measurement.

Finally, we want to point out that there has been a long-standing discussion about the inaccuracy of the numerical simulation with an ion trap between the boundary element method (BEM) and finite element method (FEM) [41,42,43,44,45,46,47]. Unfortunately, both methods have limitations, and although we used BEM-based software Charged Particle Optics (CPO Ltd.), the maximum allowed number of electrode segments was limited, and therefore only part of the entire electrode layout could be included in the numerical model as shown in Figure 2. We believe that this limitation incurs the small difference of approximately 7% between the measured and calculated axial secular frequency shown in Figure 11, which should be independent of RF voltage accuracy in the first-order approximation. Slight non-linearity between the numerical estimation of the first radial frequency and the voltage used in the calculation in Figure 11 is another evidence that the simulation result cannot be used as an absolute reference to evaluate the accuracy of our measurement. Note that, although we might not know the absolute value of the amplified voltage, we know the relative ratio among the applied voltages in each measurement (from Equation (1)), and our measurement shows that it is linear within the measured range. Thus, we claim that the major contribution of the discrepancy between the two voltages in Figure 11 comes from numerical simulation limitations rather than from the inaccuracy of our proposed measurement method.

Although the accuracy of the numerical simulation can be improved with the development of new tools [47], as the demand for scalable quantum computing based on ion traps increases, accurate modeling of surface ion traps remains still challenging because of the complexity of the chip design [48] and the small feature size compared to the entire chip. Moreover, when the absolute RF voltage is unknown, there is ambiguity in determining the accuracy of the tool. Therefore, our method to measure the absolute voltage of the amplified RF voltage will serve as an essential tool to cross-check the accuracy of the numerical simulation.

## 4. Discussion and Conclusions 

In this study, we proposed measuring the absolute amplitude of large RF voltages applied to ion traps using a modularized voltage divider. The frequency response of the voltage divider module is first characterized independent of the helical resonator, and the characterization data are used to convert measured divided voltages to amplified voltage applied to the trap. We compared the amplitude of the RF voltage applied to the ion trap measured by this method with amplitudes estimated by the measured secular frequencies and numerical simulation. The measured functional relation and the prediction from the numerical simulation showed the difference within 13% in the worst case. This comparison showed only a small discrepancy, which might come from the inaccuracy of the numerical simulation. Therefore, the proposed method can be used to directly measure RF high voltages applied to an ion trap without any fitting parameters, and in principle, it can be applied to ion trap experiments including various types of RF resonators, if proper modifications are made.

Finally, our method can help improve the accuracy of numerical simulation tools for ion traps by providing the relation between measured secular frequencies and absolute RF voltages.

## Data Availability

Data are contained within the article.
